# Supplementary figures and images for: Mam-Incept-Net: a novel inception model for precise interpretation of mammography images
Source: PeerJ Comput Sci. 2025 Aug 28;11:e3149. doi: 10.7717/peerj-cs.3149 (PMC12453803; doi:10.7717/peerj-cs.3149)

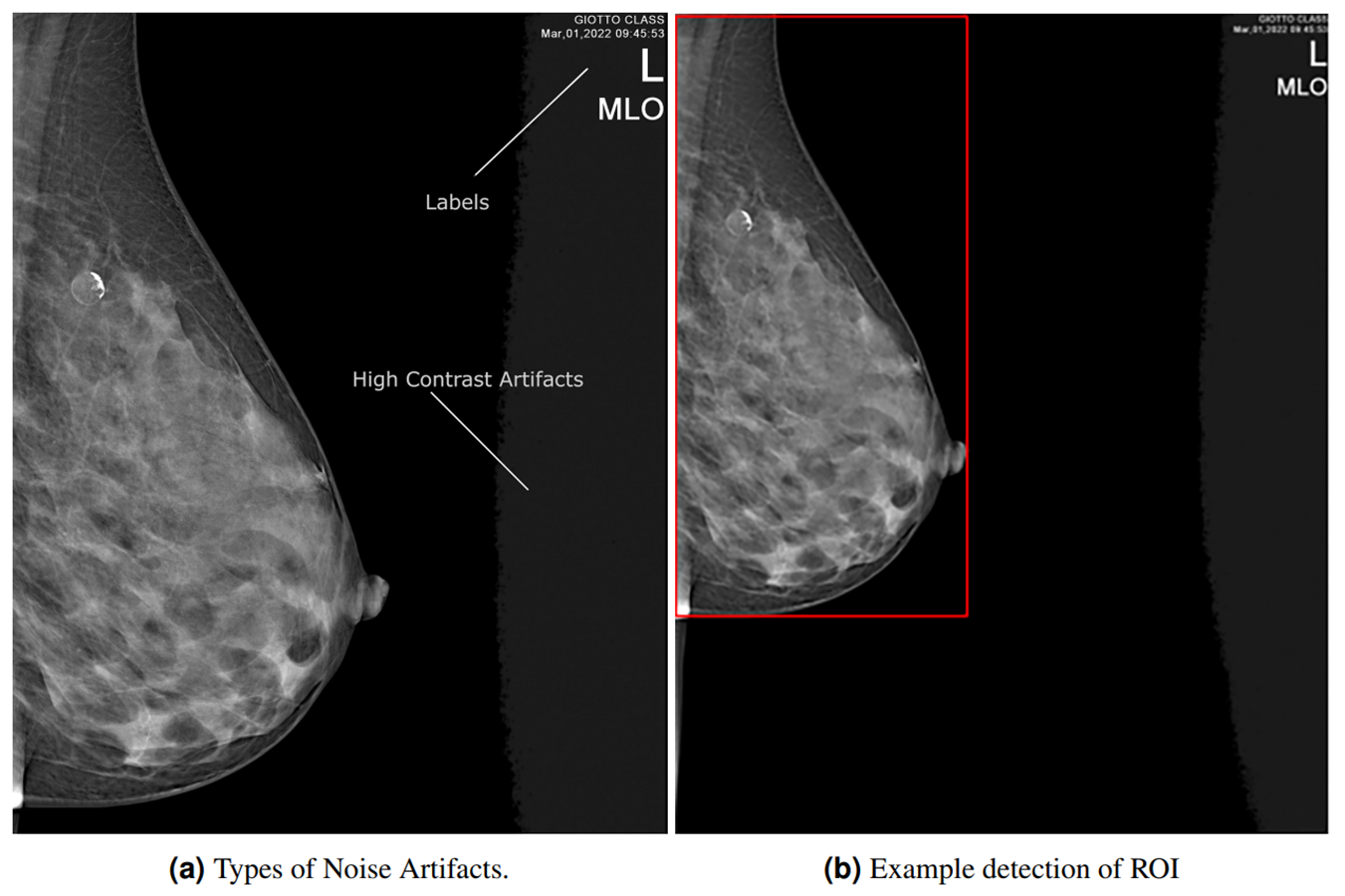

Supplement: Supplemental Information 3 [file peerj-cs-11-3149-s003.png]

# Learning Rate Schedule (for fine tuning)

Learning Rate

$1e-5$

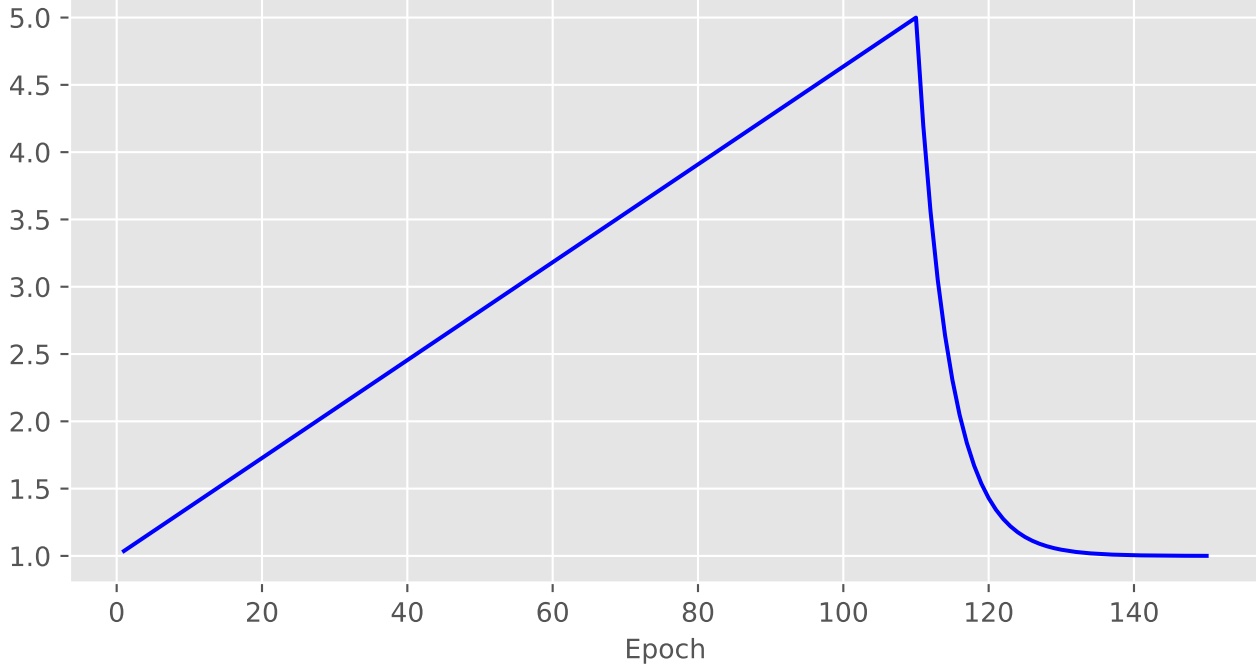

Supplement: Supplemental Information 4 [file peerj-cs-11-3149-s004.pdf]

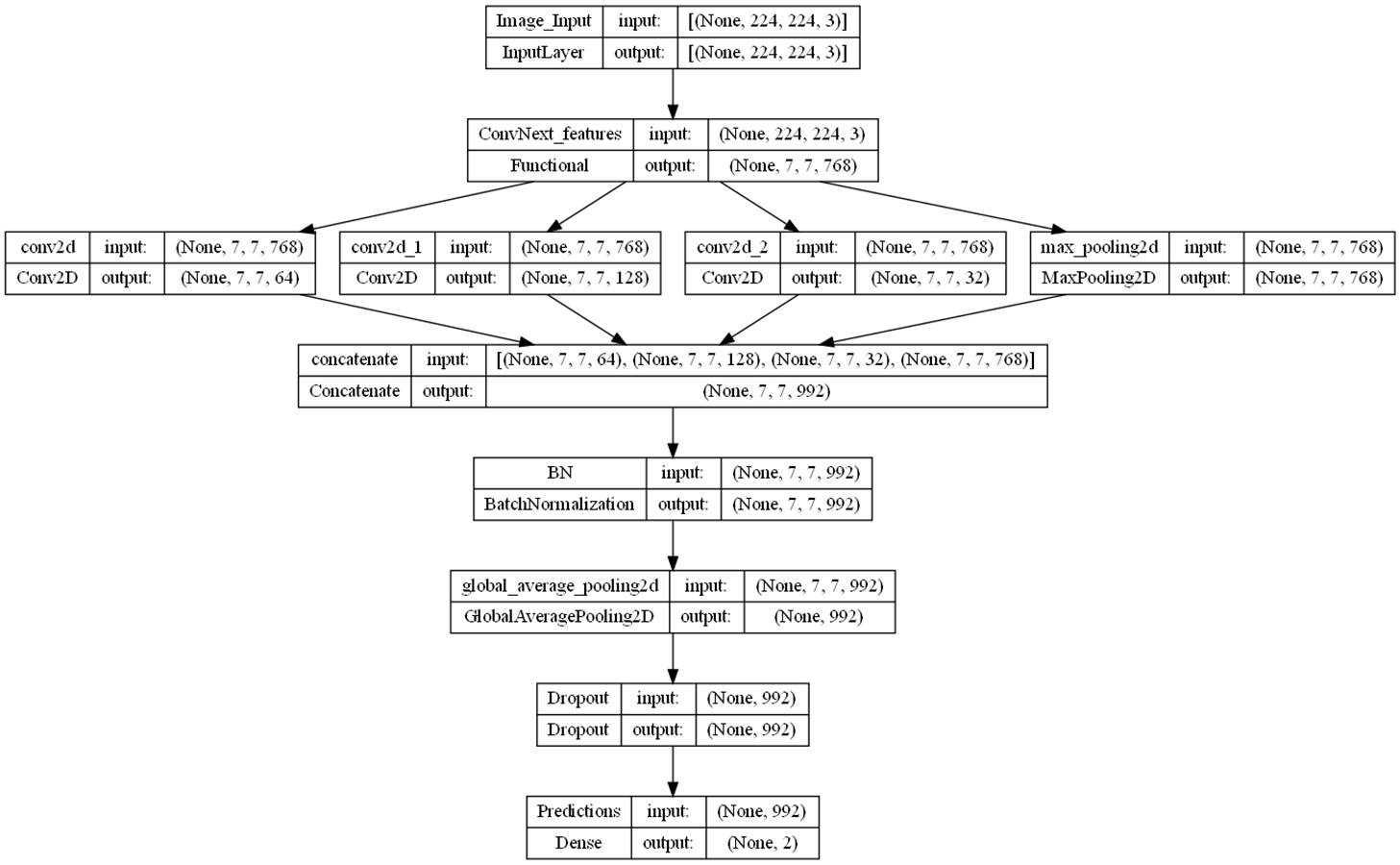

Supplement: Supplemental Information 5 [file peerj-cs-11-3149-s005.png]
